# A Novel Ferroptosis-related Gene Signature for Overall Survival Prediction in Patients with Hepatocellular Carcinoma

**DOI:** 10.7150/ijbs.45050

**Published:** 2020-07-06

**Authors:** Jie-ying Liang, De-shen Wang, Hao-cheng Lin, Xiu-xing Chen, Hui Yang, Yun Zheng, Yu-hong Li

**Affiliations:** 1 State Key Laboratory of Oncology in South China, Sun Yat-sen University Cancer Center, Collaborative Innovation Center for Cancer Medicine, Guangzhou, People's Republic of China; 2 Department of Medical Oncology, Sun Yat-sen University Cancer Center, Guangzhou, People's Republic of China; 3 Department of Hepatobiliary Oncology, Sun Yat-Sen University Cancer Center, Guangzhou, China

**Keywords:** hepatocellular carcinoma, ferroptosis, gene signature, overall survival, immune status

## Abstract

Hepatocellular carcinoma (HCC) is a highly heterogeneous disease, which makes the prognostic prediction challenging. Ferroptosis, an iron-dependent form of regulated cell death, can be induced by sorafenib. However, the prognostic value of ferroptosis-related genes in HCC remains to be further elucidated. In this study, the mRNA expression profiles and corresponding clinical data of HCC patients were downloaded from public databases. The least absolute shrinkage and selection operator (LASSO) Cox regression model was utilized to construct a multigene signature in the TCGA cohort. HCC patients from the ICGC cohort were used for validation. Our results showed that most of the ferroptosis-related genes (81.7%) were differentially expressed between HCC and adjacent normal tissues in the TCGA cohort. Twenty-six differentially expressed genes (DEGs) were correlated with overall survival (OS) in the univariate Cox regression analysis (all adjusted *P*< 0.05). A 10-gene signature was constructed to stratify patients into two risk groups. Patients in the high-risk group showed significantly reduced OS compared with patients in the low-risk group (*P* < 0.001 in the TCGA cohort and *P* = 0.001 in the ICGC cohort). The risk score was an independent predictor for OS in multivariate Cox regression analyses (HR> 1, *P*< 0.01). Receiver operating characteristic (ROC) curve analysis confirmed the signature's predictive capacity. Functional analysis revealed that immune-related pathways were enriched, and immune status were different between two risk groups. In conclusion, a novel ferroptosis-related gene signature can be used for prognostic prediction in HCC. Targeting ferroptosis may be a therapeutic alternative for HCC.

## Introduction

Liver cancer ranks sixth in terms of incidence among malignancies and is the fourth leading cause of tumor-related death worldwide 1. Hepatocellular carcinoma (HCC), the most prevalent type of primary liver cancer, is correlated with several well-known etiologies, including chronic HBV or HCV infection, alcohol abuse, nonalcoholic fatty liver disease, and exposure to dietary toxins such as aflatoxins 2. HCC is a highly heterogeneous disease that has been documented at interpatient, intertumoral and intratumoral level 3-5. The overall survival of patients with HCC varies significantly across the world 2, with a 5-year survival rate of only 18% in the United States 6. The complex etiologic factors, along with the high-level heterogeneity of HCC, make prognostic prediction challenging. Furthermore, considering the limited treatment strategies for HCC, there is an additional need for the development of novel prognostic models.

Ferroptosis is an iron-dependent form of regulated cell death (RCD) that is driven by the lethal accumulation of lipid peroxidation 7, 8. In recent years, the induction of ferroptosis has emerged as a promising therapeutic alternative to trigger cancer cell death, especially for malignancies that are resistant to traditional treatments 9, 10. Apart from ferroptosis-inducing agents, numerous genes have also been identified as modulators or markers of ferroptosis. Previous studies have reported that ferroptosis plays a vital role in HCC, and some genes, such as CISD1 11 and the polymorphism of the TP53 gene (S47 variant) 12, are known to regulate ferroptosis negatively. On the other hand, other ferroptosis-related genes, such as Rb 13, NRF2 14 and MT1G 15, might protect HCC from sorafenib-induced ferroptosis. However, whether these ferroptosis-related genes are correlated with HCC patient prognosis remains largely unknown.

In the present study, we first downloaded mRNA expression profiles and corresponding clinical data of HCC patients from public databases. Then, we constructed a prognostic multigene signature with ferroptosis-related differentially expressed genes (DEGs) in the TCGA cohort and validated it in the ICGC cohort. Finally, we further performed functional enrichment analysis to explore the underlying mechanisms.

## Materials and Methods

### Data collection

#### TCGA-LIHC cohort and ICGC (LIRI-JP) cohort

The level 3 RNA sequencing (RNA-seq) data and corresponding clinical information of 371 HCC patients were downloaded from the TCGA website up to November 15, 2019 (https://portal.gdc.cancer.gov/repository). The gene expression profiles were normalized using the scale method provided in the "limma" R package. RNA-seq data and clinical information of another 231 tumor samples were obtained from the ICGC portal (https://dcc.icgc.org/projects/LIRI-JP). These samples were primarily derived from a Japanese population with HBV or HCV infection 16. Normalized read count values were used. The data from TCGA and ICGC are both publicly available. Thus, the present study was exempted from the approval of local ethics committees. The current research follows the TCGA and ICGC data access policies and publication guidelines.

Then, 60 ferroptosis-related genes were retrieved from the previous literature 8, 9, 17, 18 and are provided in Supplementary Table S1.

### Construction and validation of a prognostic ferroptosis-related gene signature

The "limma" R package was used to identify the differentially expressed genes (DEGs) between tumor tissues and adjacent nontumorous tissues with a false discovery rate (FDR) < 0.05 in the TCGA cohort. Univariate Cox analysis of overall survival (OS) was performed to screen ferroptosis-related genes with prognostic values. *P* values were adjusted by Benjamini & Hochberg (BH) correction. An interaction network for the overlapping prognostic DEGs was generated by the STRING database (version 11.0) 19. To minimize the risk of overfitting, the LASSO-penalized Cox regression analysis was applied to construct a prognostic model 20, 21. The LASSO algorithm was used for variable selection and shrinkage with the "glmnet" R package. The independent variable in the regression was the normalized expression matrix of candidate prognostic DEGs, and the response variables were overall survival and status of patients in the TCGA cohort. Penalty parameter (λ) for the model was determined by tenfold cross-validation following the minimum criteria (i.e. the value of λ corresponding to the lowest partial likelihood deviance). The risk scores of the patients were calculated according to the normalized expression level of each gene and its corresponding regression coefficients. The formula was established as follows: score= e^sum (each gene's expression × corresponding coefficient)^. The patients were stratified into high-risk and low-risk groups based on the median value of the risk score. Based on the expression of genes in the signature, PCA was carried out with the "prcomp" function of the "stats" R package. Besides, t-SNE were performed to explore the distribution of different groups using the "Rtsne" R package. For the survival analysis of each gene, the optimal cut-off expression value was determined by the "surv_cutpoint" function of the "survminer" R package. The "survivalROC" R package was used to conduct time‐dependent ROC curve analyses to evaluate the predictive power of the gene signature.

### Functional enrichment analysis

The "clusterProfiler" R package was utilized to conduct Gene Ontology (GO) and Kyoto Encyclopedia of Genes and Genomes (KEGG) analyses based on the DEGs (|log2FC| ≥ 1, FDR < 0.05) between the high-risk and low-risk groups. *P* values were adjusted with the BH method. The infiltrating score of 16 immune cells and the activity of 13 immune-related pathways were calculated with single-sample gene set enrichment analysis (ssGSEA) 22 in the "gsva" R package. The annotated gene set file is provided in Supplementary Table S2.

### Statistical analysis

Student's t-test was used to compare gene expression between tumor tissues and adjacent nontumorous tissues. Differences in proportions were compared by the Chi-squared test. Mann-Whitney test with *P* values adjusted by the BH method was used to compare the ssGSEA scores of immune cells or pathways between the high risk and low risk group. The OS between different groups was compared by Kaplan-Meier analysis with the log-rank test. Univariate and multivariate Cox regression analyses were implemented to identify independent predictors of OS. All statistical analyses were performed with R software (Version 3.5.3) or SPSS (Version 23.0). If not specified above, a *P* value less than 0.05 was considered statistically significant, and all *P* values were two-tailed.

## Results

**Figure 1:**
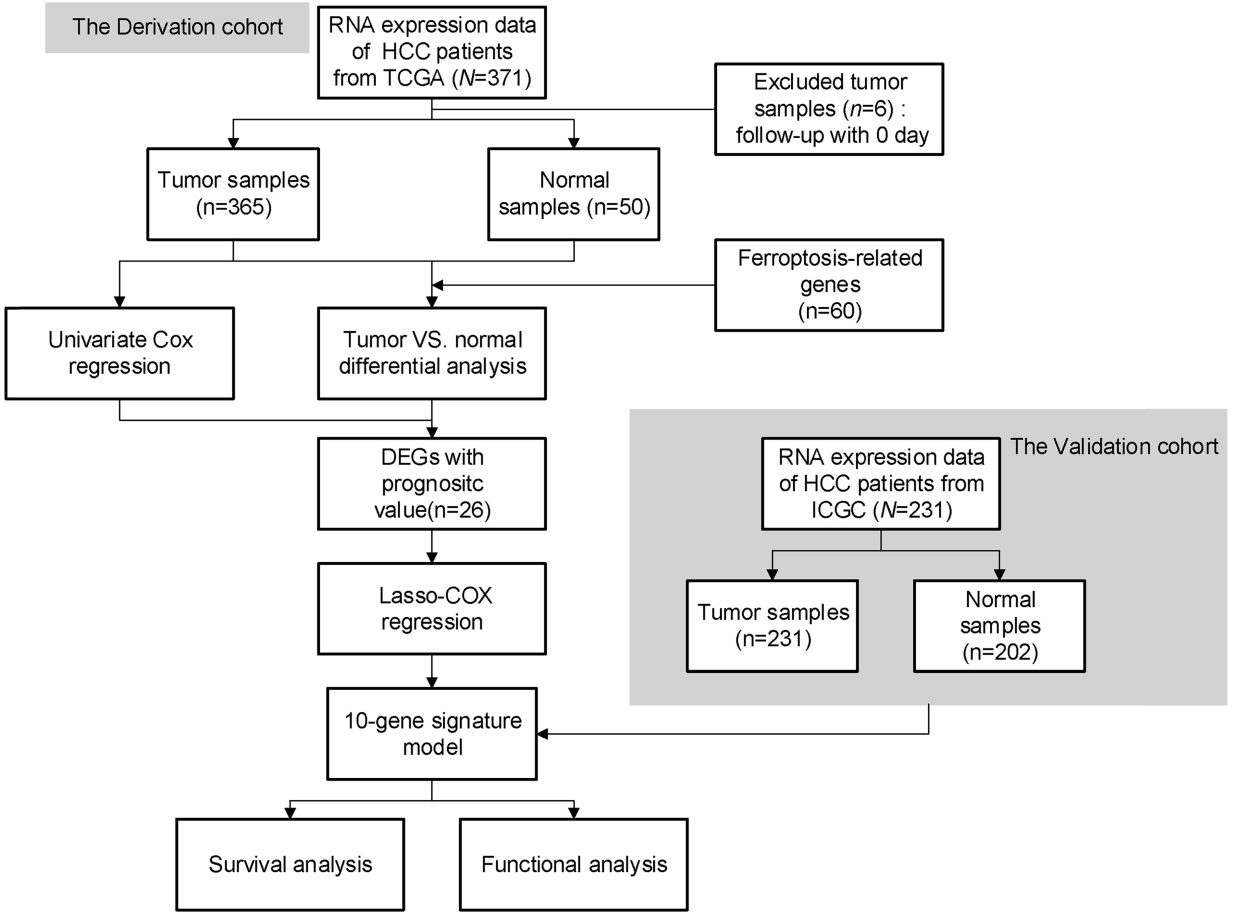
Flow chart of data collection and analysis.

The flow chart of this study is shown in Fig. 1. A total of 365 HCC patients from the TCGA-LIHC cohort and 231 HCC patients from the ICGC (LIRI-JP) cohort were finally enrolled. The detailed clinical characteristics of these patients are summarized in Table 1.

### Identification of prognostic ferroptosis-related DEGs in the TCGA cohort

**Figure 2:**
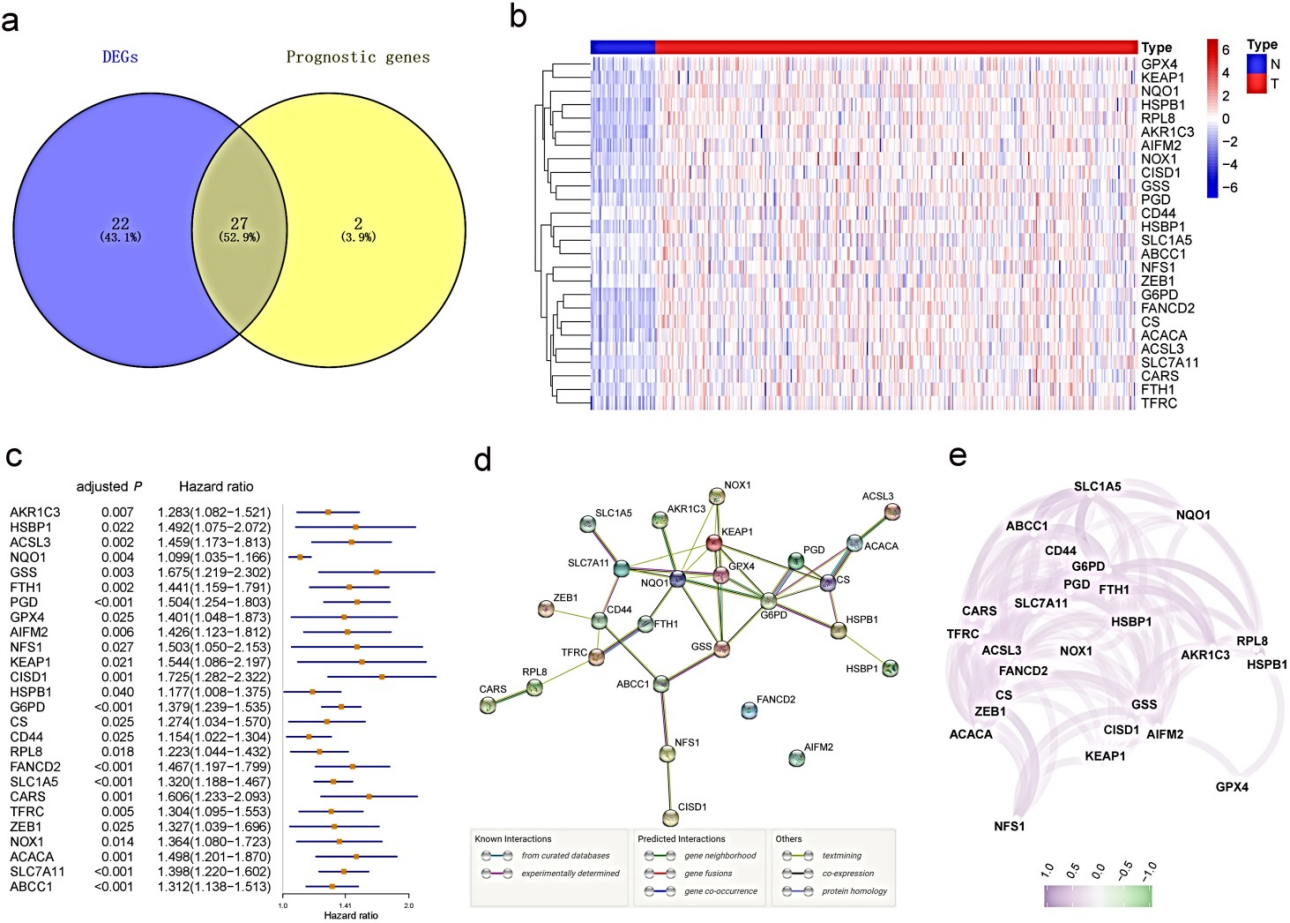
** Identification of the candidate ferroptosis-related genes in the TCGA cohort. a.** Venn diagram to identify differentially expressed genes between tumor and adjacent normal tissue that were correlated with OS. **b.** The 26 overlapping genes were all upregulated in tumor tissue. **c.** Forest plots showing the results of the univariate Cox regression analysis between gene expression and OS. **d.** The PPI network downloaded from the STRING database indicated the interactions among the candidate genes. **e.** The correlation network of candidate genes. The correlation coefficients are represented by different colors.

Most of the ferroptosis-related genes (49/60, 81.7%) were differentially expressed between tumor tissues and adjacent nontumorous tissues, and 27 of them were correlated with OS in the univariate Cox regression analysis (Fig. 2a). Unreasonably, HMOX1 was upregulated in tumor samples, but its expression predicted an excellent prognosis in the univariate Cox analysis, so it was excluded from further study. A total of 26 prognostic ferroptosis-related DEGs were preserved (all FDR <0.05, Fig.2b-c). The interaction network among these genes indicated that GPX4, G6PD and NQO1 were the hub genes (Fig.2d). The correlation between these genes is presented in Fig. 2e.

### Construction of a prognostic model in the TCGA cohort

**Figure 3:**
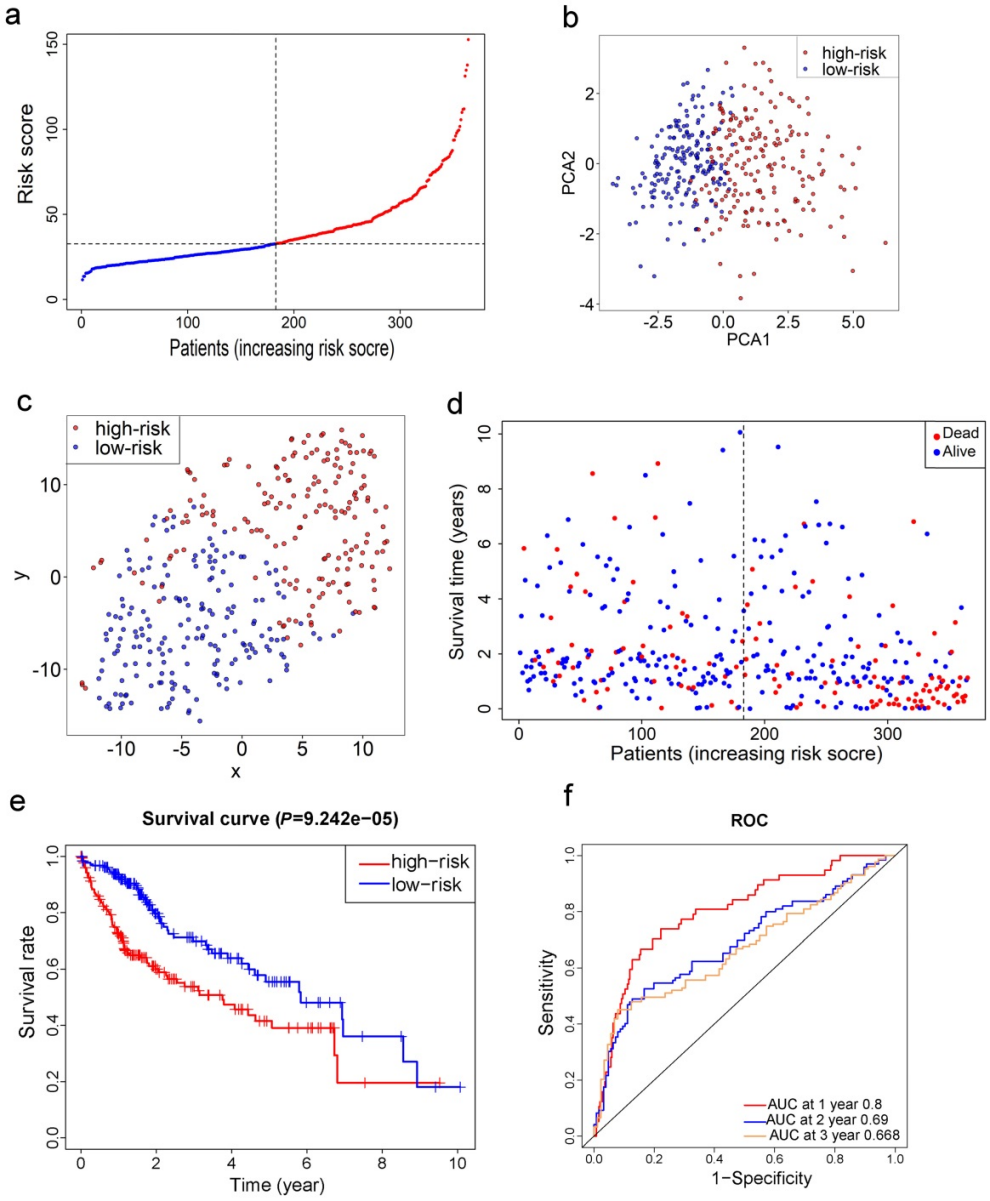
** Prognostic analysis of the 10-gene signature model in the TCGA cohort. a.** The distribution and median value of the risk scores in the TCGA cohort. **b.** PCA plot of the TCGA cohort. **c.** t-SNE analysis of the TCGA cohort. **d.** The distributions of OS status, OS and risk score in the TCGA cohort. **e.** Kaplan-Meier curves for the OS of patients in the high-risk group and low-risk group in the TCGA cohort. **f.** AUC of time-dependent ROC curves verified the prognostic performance of the risk score in the TCGA cohort.

LASSO Cox regression analysis was applied to establish a prognostic model using the expression profile of the 26 genes mentioned above. A 10-gene signature was identified based on the optimal value of λ (Fig. S1). Survival analyses, according to the optimal cut-off expression value of each gene, indicated that high expression of these genes all correlated with a poor prognosis (all adjusted *P*<0.05, Fig. S2). The risk score was calculated as follows: e ^(0.105 * expression level of SLC7A11 + 0.116 * expression level of G6PD + 0.106 * expression level of CISD1 + 0.076 * expression level of CARS + 0.077 * expression level of SLC1A5 + 0.092 * expression level of ACACA + 0.005 * expression level of ACSL3 + 0.006 * expression level of NQO1 + 0.087 * expression level of NFS1 + 0.135 * expression level of GPX4)^. The patients were stratified into a high-risk group (n=182) or a low-risk group (n=183) according to the median cut-off value (Fig. 3a). The higher risk group was found to be significantly associated with higher tumor grade, higher AFP, a higher rate of vascular invasion and advanced TNM stage in the TCGA cohort (Table 2). PCA and t-SNE analysis indicated the patients in different risk groups were distributed in two directions (Fig. 3b-c). As shown in Fig. 3d, patients with high risk had a higher probability of death earlier than those with low risk. Consistently, the Kaplan-Meier curve indicated that patients in the high-risk group had a significantly worse OS than their low-risk counterparts (Fig. 3e, *P*< 0.001). The predictive performance of the risk score for OS was evaluated by time-dependent ROC curves, and the area under the curve (AUC) reached 0.800 at 1 year, 0.690 at 2 years, and 0.668 at 3 years (Fig. 3f).

### Validation of the 10-gene signature in the ICGC cohort

**Figure 4:**
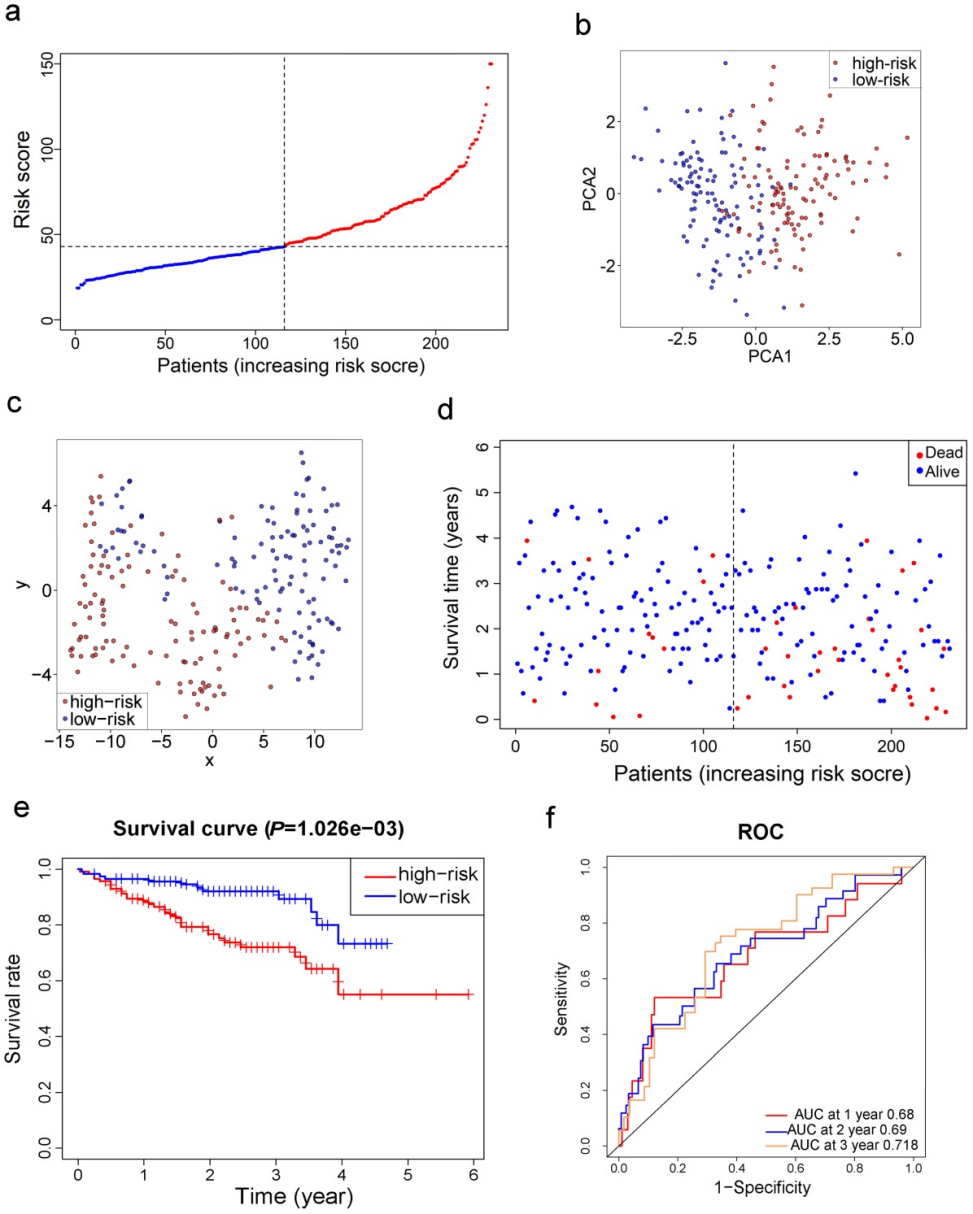
** Validation of the 10-gene signature in the ICGC cohort. a.** The distribution and median value of the risk scores in the ICGC cohort. **b.** PCA plot of the ICGC cohort. **c.** t-SNE analysis of the ICGC cohort. **d.** The distributions of OS status, OS and risk score. **e.** Kaplan-Meier curves for the OS of patients in the high-risk group and low-risk group. **f.** AUC of time-dependent ROC curves in the ICGC cohort.

Survival analyses of ten genes in the signature confirmed that these genes correlated with poor OS in the ICGC cohort except for CARS (all adjusted *P*< 0.05, Fig. S3). To test the robustness of the model constructed from the TCGA cohort, the patients from the ICGC cohort were also categorized into high- or low-risk groups by the median value calculated with the same formula as that from the TCGA cohort. The high risk group was also correlated with advanced TNM stage in the ICGC cohort (Table 2). Similar to the results obtained from the TCGA cohort, PCA and t-SNE analysis confirmed that patients in two subgroups were distributed in discrete directions (Fig. 4b-c). Likewise, patients in the high-risk group were more likely to encounter death earlier (Fig. 4d) and had a reduced survival time compared with those in the low-risk group (Fig. 4e, *P*= 0.001). Besides, the AUC of the 10-gene signature was 0.680 at 1 year, 0.690 at 2 years, and 0.718 at 3 years (Fig. 4f).

### Independent prognostic value of the 10-gene signature

**Figure 5:**
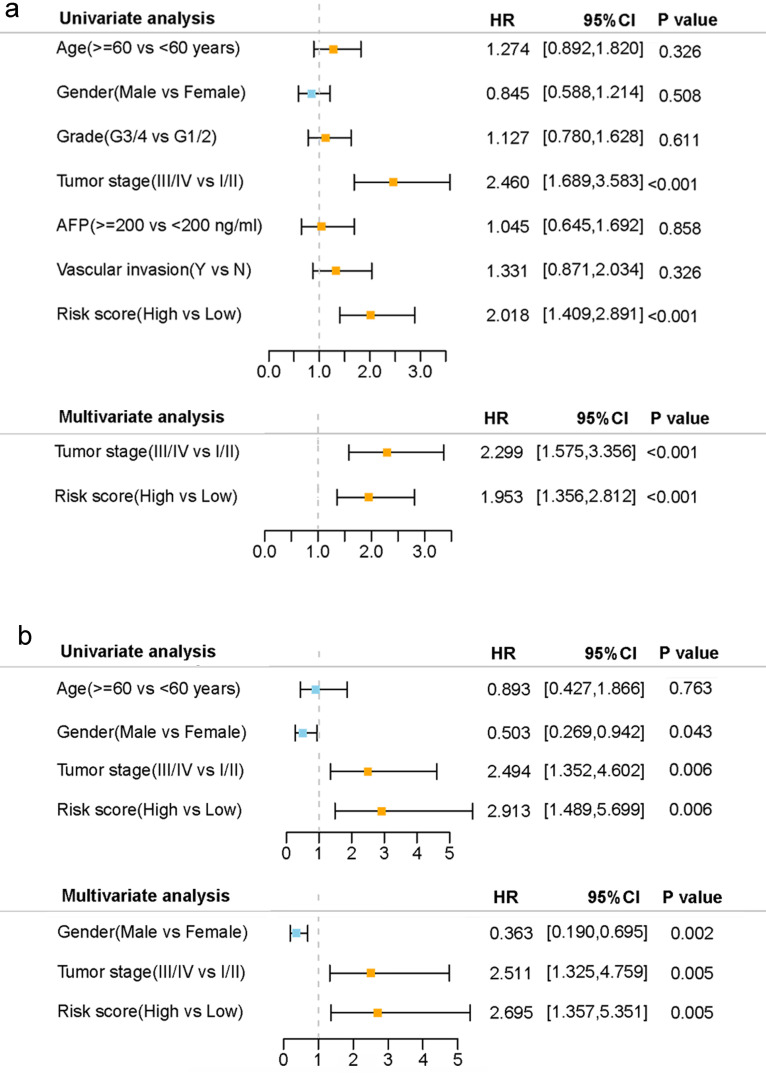
Results of the univariate and multivariate Cox regression analyses regarding OS in the TCGA derivation cohort (a) and the ICGC validation cohort (b).

Univariate and multivariate Cox regression analyses were carried out among the available variables to determine whether the risk score was an independent prognostic predictor for OS. In univariate Cox regression analyses, the risk score was significantly associated with OS in both the TCGA and the ICGC cohort (HR= 2.018, 95% CI = 1.409-2.891, *P*< 0.001; HR= 2.913, 95% CI = 1.489-5.699, *P*= 0.006, respectively) (Fig. 5a, b). After correction for other confounding factors, the risk score still proved to be an independent predictor for OS in the multivariate Cox regression analysis (TCGA cohort: HR =1.953, 95% CI = 1.356-2.812, *P*<0.001; ICGC cohort: HR=2.695, 95% CI = 1.357-5.351, *P* = 0.005; Fig. 5a, b).

### Functional analyses in the TCGA and the ICGC cohort

**Figure 6:**
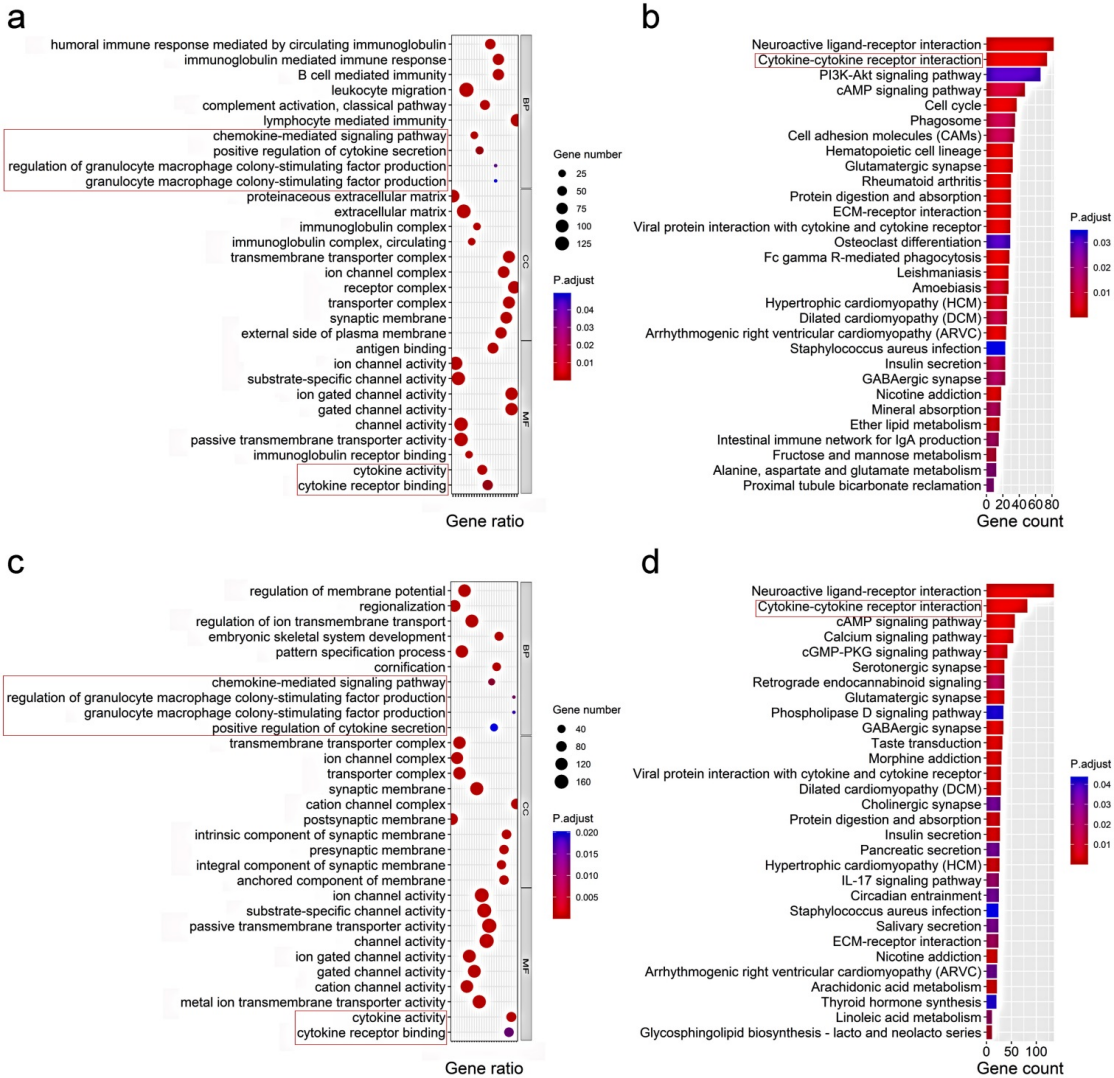
**Representative results of GO (a, c) and KEGG analyses (b, d) .** The most significant or shared GO enrichment and KEGG pathways in the TCGA cohort (**a, b**) and ICGC cohort **(c, d)** are displayed. The pink rectangles indicate the immune-related pathways that are overlapped between the two cohorts.

To elucidate the biological functions and pathways that were associated with the risk score, the DEGs between the high-risk and low-risk groups were used to perform GO enrichment and KEGG pathway analyses. As expected, DEGs were enriched in several iron-related molecular functions, such as ion channel activity and ion gated channel activity in both the TCGA and ICGC cohort (*P*. adjust < 0.05, Fig. 6a, c). Interestingly, the DEGs from the TCGA cohort were also obviously enriched in many immune-related biological processes (*P*. adjust < 0.05, Fig. 6a). Six immune-related biological processes or molecular functions were validated by the ICGC cohort, including chemokine-mediated signaling pathway, positive regulation of cytokine secretion, regulation of granulocyte macrophage colony-stimulating factor production, granulocyte macrophage colony-stimulating factor production, cytokine activity, and cytokine receptor binding (*P*. adjust < 0.05, Fig. 6c). KEGG pathway analyses also indicated that the cytokine-cytokine receptor interaction pathway was enriched in both cohorts (*P*. adjust < 0.05, Fig. 6b, d).

**Figure 7:**
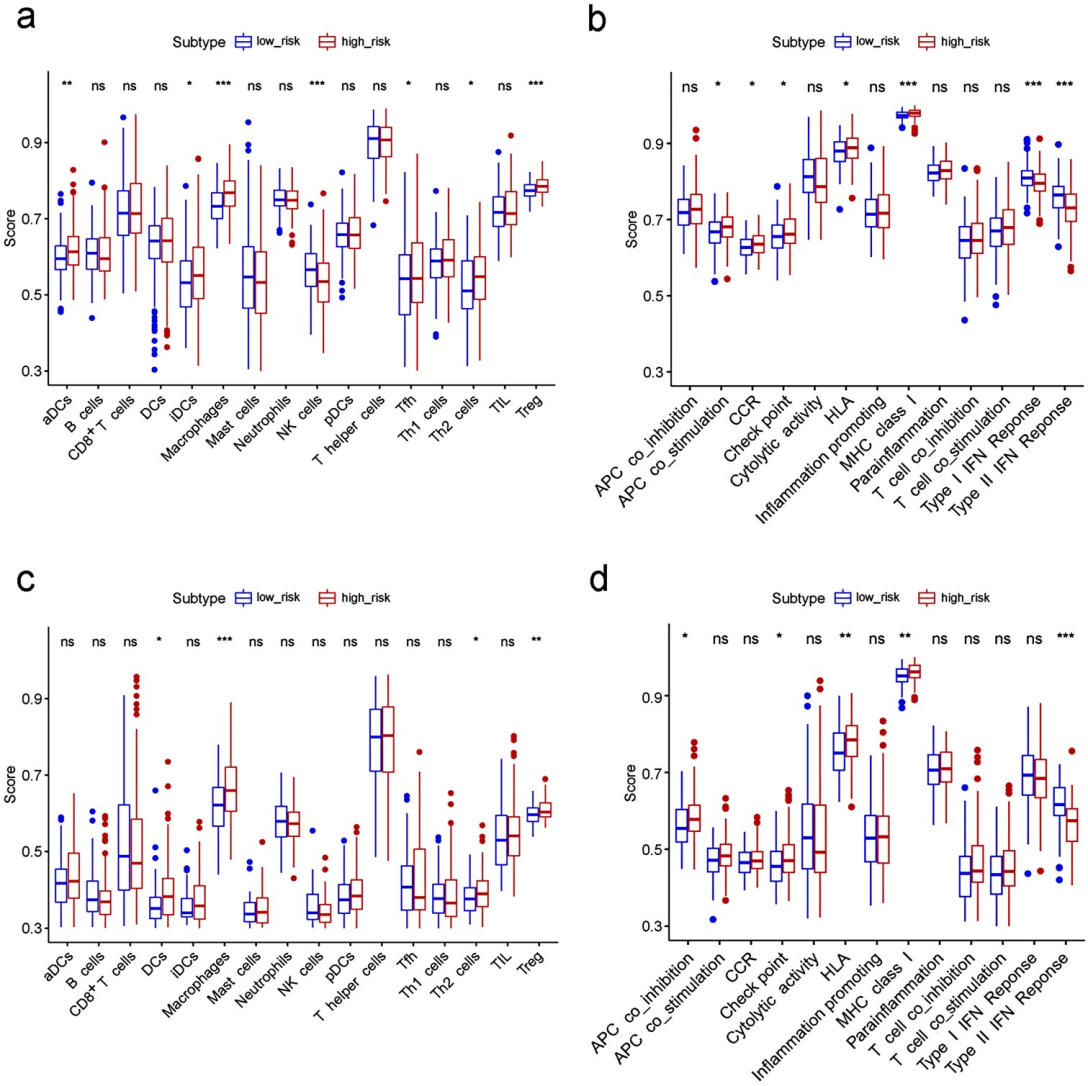
** Comparison of the ssGSEA scores between different risk groups in the TCGA cohort (a, b) and ICGC cohort (c, d).** The scores of 16 immune cells **(a, c)** and 13 immune-related functions** (b, d)** are displayed in boxplots. CCR, cytokine-cytokine receptor. Adjusted *P* values were showed as: ns, not significant; *, *P*< 0.05; **, *P*< 0.01; ***, *P*< 0.001.

To further explore the correlation between the risk score and immune status, we quantified the enrichment scores of diverse immune cell subpopulations, related functions or pathways with ssGSEA. Interestingly, contents of the antigen presentation process, including the score of aDCs, iDCs, APC co-stimulation, HLA and MHC class I, were significantly different between the low risk and high risk group in the TCGA cohort (all adjusted *P*< 0.05, Fig. 7a-b). The cytokine-cytokine receptor interaction that was enriched in the KEGG analyses had a higher score in the high risk group of the TCGA cohort (adjusted *P*< 0.05, Fig. 7b). Moreover, the score of type II IFN response, type I IFN response, and NK cells were lower in the high risk group, while the activity of checkpoint molecules, the scores of macrophages or Treg cells were just the opposite (adjusted *P*< 0.05, Fig. 7a-b). Comparisons in the ICGC cohort confirmed the differences of HLA, MHC class I, type II IFN response, checkpoint molecules, macrophages and Treg cells between two risk groups (adjusted *P*< 0.05, Fig. 7c-d). In particular, the scores of macrophages were the most statistically different between the two risk groups in both the TCGA and the ICGC cohort, which was consistent with the findings in the GO analysis.

## Discussion

In the current study, we systematically investigated the expression of 60 ferroptosis-related genes in HCC tumor tissues and their associations with OS. A novel prognostic model integrating 10 ferroptosis-related genes was firstly constructed and validated in an external cohort. Functional analyses revealed that immune-related pathways were enriched.

Although a few previous studies 13-15 have indicated that several genes might regulate drug-induced ferroptosis in HCC, their correlation with HCC patients' OS remains largely unknown. To our surprise, most of the ferroptosis-related genes (81.7%) were differentially expressed between tumor and adjacent nontumorous tissues, and more than half of them were correlated with OS in the univariate Cox regression analysis. These results significantly indicated the potential role of ferroptosis in HCC and the possibility of building a prognostic model with these ferroptosis-related genes.

The prognostic model proposed in the present study was composed of 10 ferroptosis-related genes (ACACA, ACSL3, CISD1, CARS, G6PD, GPX4, NQO1, NFS1, SLC7A11, SLC1A5). These genes could be roughly classified into four categories, including iron metabolism (NFS1, CISD1), lipid metabolism (ACACA, ACSL3, GPX4), (anti)oxidant metabolism (CARS, NQO1, SLC7A11) and energy metabolism (G6PD, SLC1A5) 9. NFS1, an enzyme involved in synthesizing iron-sulfur clusters using sulfur from cysteine, protects cells from ferroptosis in lung cancer 23. The genetic inhibition of CISD1 results in iron accumulation and subsequent oxidative injury in the mitochondria and thus contributes to erastin-induced ferroptosis in HCC cells 11. In terms of lipid metabolism, ACACA impacts the rate-limiting step in fatty acid synthesis and is a key regulator of tumor cell survival. Knockout of ACACA suppresses pharmacological agent-induced ferroptosis 24*.* ACSL3, which converts exogenous monounsaturated fatty acids (MUFAs) into fatty acyl-CoAs, is required for MUFA activation and promotes a ferroptosis-resistant cell status 25. GPX4 has been considered to be the primary enzyme that prevents ferroptosis for a long time due to its role in converting lipid hydroperoxides into nontoxic lipid alcohols 26. For (anti)oxidant metabolism, knockdown of CARS inhibits erastin-induced ferroptosis by preventing the induction of lipid reactive oxygen in fibrosarcoma cells 27. In contrast, knockdown of NQO1 enhances erastin and sorafenib-induced ferroptosis in HCC cells 14. Similarly, knockdown of SLC7A11, a subunit of system Xc to import cystine in the cell, sensitized fibrosarcoma cells to erastin-induced death 7. G6PD and SLC1A5 are two ferroptosis regulators involved in energy metabolism. G6PD, which is involved in the pentose phosphate pathway, has been reported to prevent erastin-induced ferroptosis when it was knocked down in non-small cell lung cancer cells 7. Inhibition or knockdown of SLC1A5 also suppresses ferroptosis 28. In summary, six of the genes (NFS1, CISD1, ACSL3, NQO1, SLC7A11, GPX4) in the prognostic model have been reported to protect cells from ferroptosis, while the remaining four genes (ACACA, CARS, G6PD, SLC1A5) are the opposite. However, these genes were all upregulated in HCC tumor tissue and were associated with poor prognosis in the current study. Whether these genes play a role in HCC patients' prognosis by influencing the process of ferroptosis remains to be elucidated, since few related studies on these genes except for CISD1 and NQO1 have been reported.

Although the mechanisms underlying tumor susceptibility to ferroptosis have been an intense area of research in the past few years, the potential modulation between tumor immunity and ferroptosis remains elusive. Based on the DEGs between different risk groups, we performed GO analyses and unexpectedly discovered that many immune-related biological processes and pathways were enriched. It is reasonable to assume that ferroptosis may have a close connection with tumor immunity. Interestingly, the contents of the antigen presentation process were significantly different between the low risk and high risk group in this study. One possible speculation is that ferroptotic cells release distinct signals, such as lipid mediators, to attract antigen-presenting cells (APCs) to the site of ferroptotically dying cells 29. Besides, the high risk groups in both the TCGA and the ICGC cohort have higher fractions of macrophages and Treg cells. Previous studies have demonstrated that increased tumor-associated macrophages [30, 31]or Treg cells [31, 32]are related to poor prognosis in HCC patients due to their role in immune invasion. Moreover, higher risk scores correlated with impaired antitumor immunity, including the activity of the type II IFN response and type I IFN response as well as the fractions of NK cells. Therefore, attenuated antitumor immunity in patients with high risk may be an explanation for their poor prognosis.

There are several limitations of this study. First, our prognostic model was both constructed and validated with retrospective data from public databases. More prospective real-world data are warranted to verify its clinical utility. Second, the intrinsic weakness of merely considering a single hallmark to build a prognostic model was inevitable, as many prominent prognostic genes in HCC might have been excluded. In addition, it should be emphasized that the links between the risk score and immune activity have not yet been experimentally addressed.

In summary, our study defined a novel prognostic model of 10 ferroptosis-related genes. This model proved to be independently associated with OS in both the derivation and validation cohorts, providing insight into the prediction of HCC prognosis. The underlying mechanisms between ferroptosis-related genes and tumor immunity in HCC remain poorly understood and warrant further investigation.

## Supplementary Material

Supplementary figures and tables.

## Figures and Tables

**Table 1:** Clinical characteristics of the HCC patients used in this study

	TCGA cohort	LIRI-JP cohort
**No. of patients**	365	231
**Age (median, range)**	61(16-90)	69(31-89)
**Gender (%)**		
Female	119(32.6%)	61(26.4%)
Male	246(67.4%)	170(72.6%)
**AFP( ng/ml)**		
≤200	201(55.1%)	NA
>200	75(20.5%)	NA
unknown	89(24.4%)	NA
**Grade(%)**		
Grade 1	55(15.1%)	NA
Grade 2	175(47.9%)	NA
Grade 3	118(32.3%)	NA
Grade 4	12(3.3%)	NA
unknown	5(1.4%)	NA
**Vascular Invasion**		
Yes	106(29.0%)	NA
No	205(56.2%)	NA
unknown	54(14.8%)	NA
**Stage(%)**		
I	170(46.6%)	36(15.6%)
II	84(23.0%)	105(45.5%)
III	83(22.7%)	71(30.7%)
IV	4(1.1%)	19(8.2%)
unknown	24(6.6%)	0(0.0%)
**Survival status**		
OS days (median)	556	780
censored(%)	126(34.5)	42(18.2)

**Table 2:** Baseline characteristics of the patients in different risk groups

Characteristics	TCGA-LIHC cohort				ICGC-LIRP-JI cohort		
	High risk	Low risk	*P* value		High risk	Low risk	*P* value
**Gender(%)**			0.882				0.315
Female	60 (33.0)	59 (32.2)			27 (23.5)	34 (29.3)	
Male	122 (67.0)	124 (67.8)			88 (76.5)	82 (70.7)	
**Age (%)**			0.789				0.975
< 60y	81 (44.5)	84 (45.9)			22 (19.1)	22 (19.0)	
≥60y	101 (55.5)	99 (54.1)			93 (80.9)	94 (81.0)	
**Tumor grade(%)**			**<0.001**				-
G1+G2	94 (51.6)	136 (74.3)			-	-	
G3+G4	85 (46.7)	45 (24.6)			-	-	
unknown	3 (1.6)	2 (1.1)			-	-	
**AFP (%)**			**0.003**				
≤200ng/ml	84 (46.2)	117 (63.9)			-	-	
>200ng/ml	46 (25.3)	29 (15.8)			-	-	
unknown	52 (28.6)	37 (20.2)			-	-	
**Vascular invasion(%)**			**0.004**				
No	87 (47.8)	118 (64.5)			-	-	
Yes	60 (33.0)	46 (25.1)			-	-	
unknown	35 (19.2)	19 (10.4)			-	-	
**TNM stage(%)**			**0.003**				**0.001**
Ⅰ+Ⅱ	116 (63.7)	138 (75.4)			58 (50.4)	83 (71.6)	
Ⅲ+Ⅳ	57 (31.3)	30 (16.4)			57 (49.6)	33 (28.4)	
unknown	9 (4.9)	15 (8.2)			-	-	

## Abbreviations

HCC: Hepatocellular carcinoma
LASSO: Least absolute shrinkage and selection operator
TCGA: The Cancer Genome Atlas
ICGC: International Cancer Genome Consortium
DEGs: Differentially expressed genes
OS: Overall survival
ROC: Receiver operating characteristic
FDR: False discovery rate
GO: Gene Ontology
KEGG: Kyoto Encyclopedia of Genes and Genomes
ssGSEA: Single-sample gene set enrichment analysis
PCA: Principal component analysis
t-SNE: t-distributed stochastic neighbor embedding
AUC: Area under the curve
HR: Hazard ratio
CI: Confidence interval
APC: Antigen presenting cell
AFP: alpha fetoprotein
aDC: Activated dendritic cell
iDC: Immature dendritic cell
pDC: Plasmacytoid dendritic cell
Tfh: T follicular helper cell
TIL: Tumor Infiltrating Lymphocyte
HLA: Human leukocyte antigen
CCR: Cytokine-cytokine receptor
