# Supplementary material for: A Novel Ferroptosis-related Gene Signature for Overall Survival Prediction in Patients with Hepatocellular Carcinoma
Source: Int J Biol Sci. 2020 Jul 6;16(13):2430–41. doi: 10.7150/ijbs.45050 (PMC7378635; doi:10.7150/ijbs.45050)
Supplement: Supplementary file 1 — Supplementary figures and tables. [file ijbsv16p2430s1.zip › Supplementary materials/Supplementary Figures.pdf]

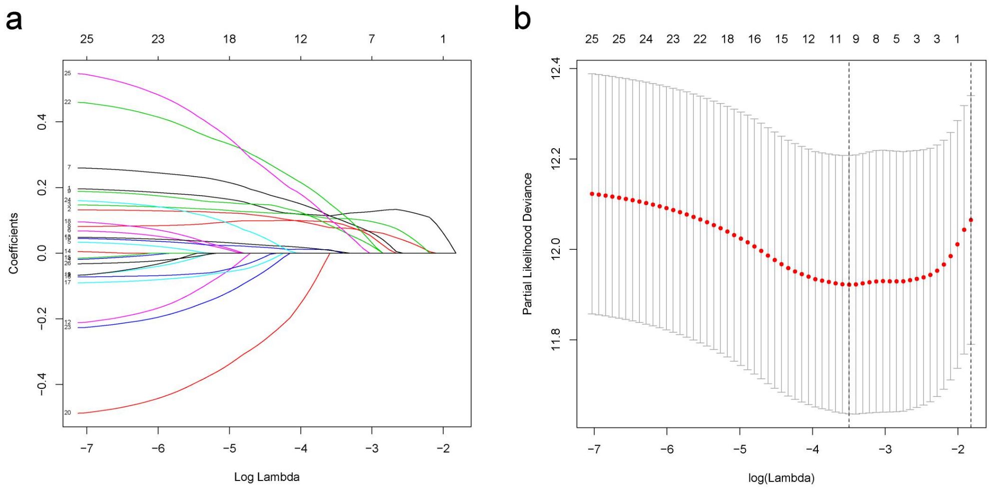

Fig. S1 Construction of a 10-gene signature model in the TCGA cohort. a. LASSO coefficient profiles of the expression of 26 candidate genes. b. Selection of the penalty parameter (λ) in the LASSO model via 10-fold cross-validation. The dotted vertical lines are plotted at the optimal values following the minimum criteria (left) and "one standard error" criteria (right).

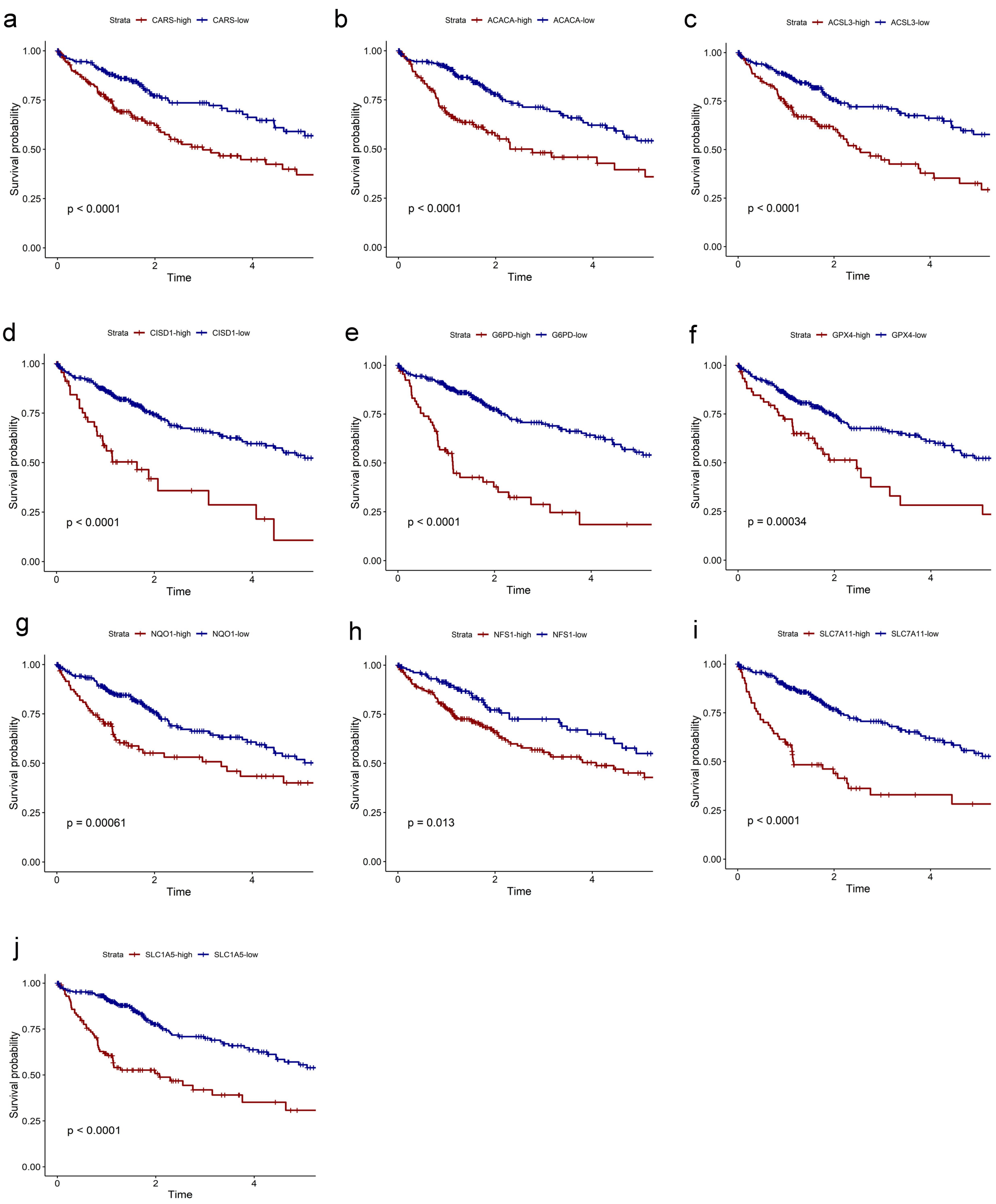

Fig. S2 Survival analyses according to the optimal cut-off expression value of each gene in the TCGA cohort. All adjusted $P < 0.05$.

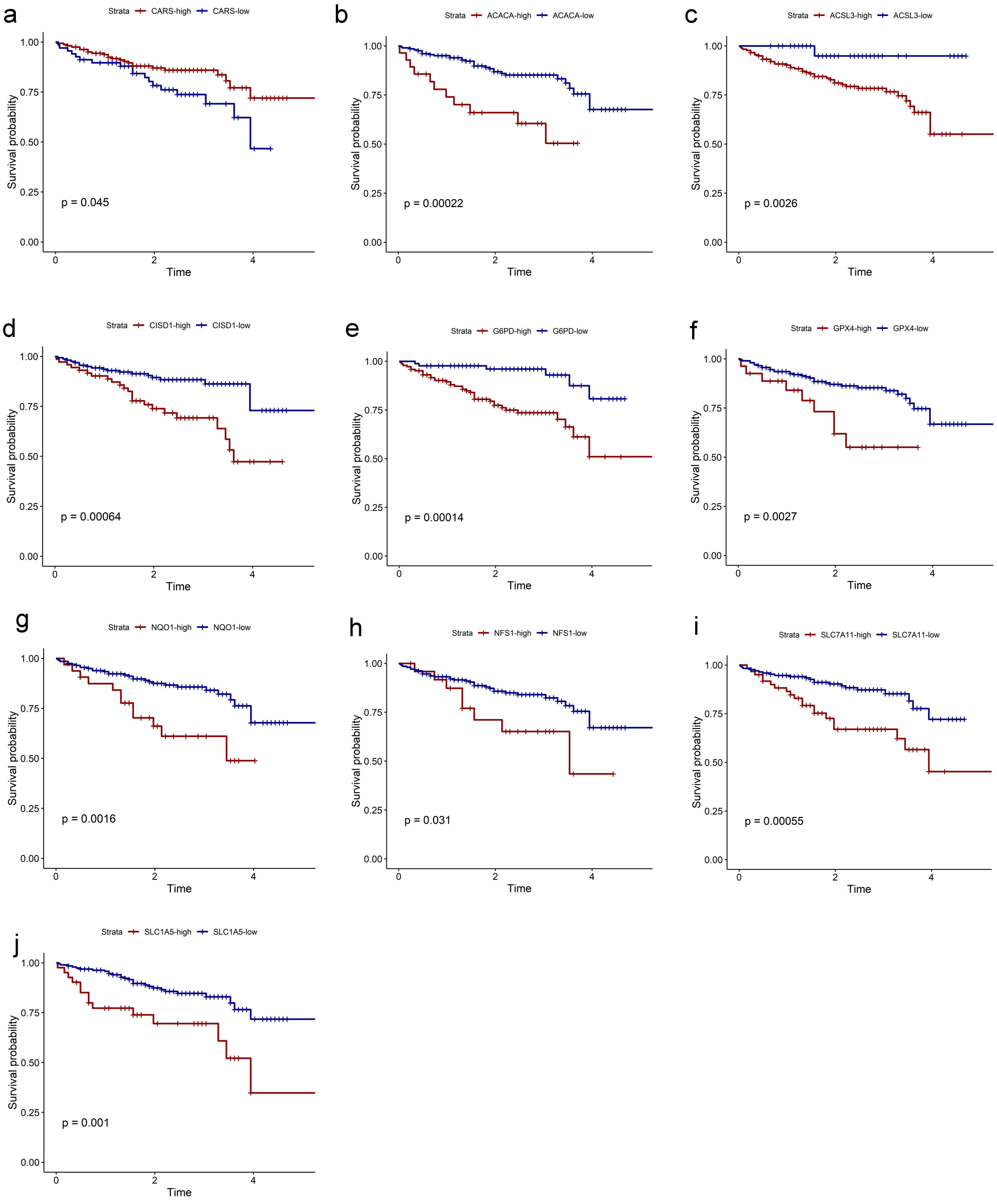

Fig. S3 Survival analyses grouped by the optimal cut-off expression value of each gene in the ICGC cohort. All adjusted $P < 0.05$.
